# Modulation of trinucleotide repeat instability by DNA polymerase β polymorphic variant R137Q

**DOI:** 10.1371/journal.pone.0177299

**Published:** 2017-05-05

**Authors:** Yaou Ren, Yanhao Lai, Eduardo E. Laverde, Ruipeng Lei, Hayley L. Rein, Yuan Liu

**Affiliations:** 1 Biochemistry Ph.D. Program, Florida International University, Miami, Florida, United States of America; 2 Department of Chemistry and Biochemistry, Florida International University, Miami, Florida, United States of America; 3 Biomolecular Sciences Institute, Florida International University, Miami, Florida, United States of America; Istituto di Genetica Molecolare, ITALY

## Abstract

Trinucleotide repeat (TNR) instability is associated with human neurodegenerative diseases and cancer. Recent studies have pointed out that DNA base excision repair (BER) mediated by DNA polymerase β (pol β) plays a crucial role in governing somatic TNR instability in a damage-location dependent manner. It has been shown that the activities and function of BER enzymes and cofactors can be modulated by their polymorphic variations. This could alter the function of BER in regulating TNR instability. However, the roles of BER polymorphism in modulating TNR instability remain to be elucidated. A previous study has shown that a pol β polymorphic variant, polβR137Q is associated with cancer due to its impaired polymerase activity and its deficiency in interacting with a BER cofactor, proliferating cell nuclear antigen (PCNA). In this study, we have studied the effect of the pol βR137Q variant on TNR instability. We showed that pol βR137Q exhibited weak DNA synthesis activity to cause TNR deletion during BER. We demonstrated that similar to wild-type pol β, the weak DNA synthesis activity of pol βR137Q allowed it to skip over a small loop formed on the template strand, thereby facilitating TNR deletion during BER. Our results further suggest that carriers with pol βR137Q polymorphic variant may not exhibit an elevated risk of developing human diseases that are associated with TNR instability.

## Introduction

Human genome is susceptible to a variety of types of DNA damage that can modify DNA bases, deoxyribose sugar phosphate (dRP) groups as well as directly break DNA backbone [1]. It has been estimated that more than 10,000 base lesions are generated per cell per day [2], and these lesions are efficiently repaired by DNA base excision repair (BER) [2, 3] through the single-nucleotide or long-patch BER subpathway [4–8].

Genome instability, typically microsatellite instability is responsible for many human diseases [9–12] including GT repeat instability that is associated with colon cancer [13] as well as trinucleotide repeat (TNR) expansion diseases [14–17].TNR expansion has been identified as the cause of more than 40 neurodegenerative diseases [14, 18] including Huntington’s disease (HD), spinocerebellar ataxia (SCA) type 1, 2, 3, 6, 17 and spinal bulbar muscular atrophy (SBMA) (Kennedy's disease) (CAG repeat expansion) [17, 19, 20], myotonic dystrophy type 1 (DM1) (CTG repeat expansion), Friedreich’s ataxia (GAA repeat expansion) and fragile X syndrome (CGG repeat expansion) [21–23]. TNR expansions can occur in both the coding or non-coding regions of the genes associated with disease development, leading to aberrant protein aggregation or deficiency of gene expression [18, 24, 25]. On the other hand, CAG repeat deletion is associated with cancer [26]. CAG repeat deletion in the androgen receptor (*AR*) gene can result in a high transcriptional activity of the AR protein [26–28], which may potentially lead to progression of prostate cancer [29, 30].

TNR instability is mediated by the formation of secondary structures including hairpins, loops and G4-quadruplex [14, 18] during DNA replication [31], repair [32, 33] and recombination [34] as well as gene transcription [35]. Furthermore, it has been shown that DNA repair [14] and gene transcription [35] play crucial roles in modulating somatic TNR instability [36] especially in postmitotic neurons. Since TNR tracts contain a stretch of guanines that are susceptible to DNA base damage [2, 37], they form hotspots of base lesions and are constantly subject to multiple cycles of BER that leads to a “toxic oxidation cycle" resulting in TNR expansion [14, 33]. This is supported by the fact that TNR expansion is promoted by increased amount of 8-oxoguanine (8-oxoG) in the neurons of HD transgenic mice [33] and germ cells of fragile X syndrome mice treated with an oxidative DNA damaging agent, potassium bromate [38]. Moreover, TNR deletion can also be induced by an alkylating DNA damaging agent, temozolomide [39] in the lymphoblasts of Friedreich’s ataxia patients through BER [40]. As a core enzyme of BER, pol β plays a critical role in maintaining genome stability [3, 41] as well as modulating TNR instability [33, 42–45]. It has been found that pol β promotes TNR expansion by performing multi-nucleotide gap-filling synthesis on a TNR repeat tract [33] and facilitating FEN1 alternate flap cleavage of a short repeat flap. This subsequently leads to ligation of a hairpin during long-patch BER [43]. It has been suggested that during DNA replication, pol β can also promote repeat expansion by extending the 3'-terminus of a hairpin to produce extra repeats [42]. A recent study has also shown that pol β can interact with mismatch repair proteins MSH2-MSH3 to promote TNR expansion [46]. On the other hand, pol β facilitates TNR deletion by skipping over a TNR hairpin on the template strand [44, 45] or bypass a 5’, 8-cyclo-2’-deoxyadenosine (cdA), a bulky base lesion located in a loop on the template of a CTG repeat tract [47]. Our previous studies have shown that pol β coordinates with FEN1 to govern the balance between the addition and removal of nucleotides during the repair on TNRs, thereby leading to TNR expansion or deletion in a damage-location dependent manner [44]. All these indicate that in coordination with FEN1, pol β modulates TNR expansion or deletion during long-patch BER via its multi-nucleotide gap-filling synthesis, strand displacement DNA synthesis, hairpin-bypass synthesis. Since the long-patch BER in the context of a TNR tract is much less efficient than the single-nucleotide BER, this allows the formation of secondary structures such as hairpins and loops. Thus, efficient BER prevents TNR instability by inhibiting DNA slippage and the formation of hairpin and loop structures in a TNR tract, whereas inefficient BER can promote the processes and TNR instability [6, 43].

Genetic variations, i.e. polymorphism of DNA repair enzymes and cofactors, 8-oxoguanine DNA glycosylase (OGG1) [48, 49], APE1 [50], X-ray repair cross-complementing protein 1 (XRCC1) [51–53], XPC [54, 55], MSH3 [56–59], RPA-CDK7 [60] among others have been reported to be associated with cancer and neurodegenerative diseases. However, some studies do not support the notion [61–63]. The controversy is due to lack of knowledge of the effects of these BER polymorphic variants on genome stability and integrity. Thus far three germline polymorphic variants of pol β with single amino acid substitution have been identified in human population. They are pol βR137Q, pol βP242R and pol βQ8R variant, which are associated with cancer [64–69]. Among them, pol βR137Q variant is particularly of interest. It contains the substitution of arginine 137 with glutamine, which occurs in the polymerase domain of pol β and is involved in mediating the interaction between pol β and proliferating cell nuclear antigen (PCNA) [70]. The pol β variant exhibits impaired DNA synthesis activity and deficiency in interacting with PCNA. This results in cellular hypersensitivity to an alkylating DNA damaging agent, methyl methanesulfonate (MMS) [64]. Thus, it is conceivable that the impaired DNA synthesis activity of pol βR137Q may disrupt the coordination between pol β and other BER proteins, promoting genome instability such as TNR instability during BER. To test this possibility, we initially characterized DNA synthesis activity of the polymorphic pol βR137Q variant and its effects on CAG and CTG repeat instability during BER. We found that pol βR137Q variant showed weaker DNA synthesis activity than wild-type pol β during BER in the context of CAG and CTG repeats. Yet, it exhibited similar ability as wild-type pol β to cause deletion of CAG and CTG repeats during BER of an abasic lesion at various locations. We provide the first evidence that pol βR137Q variant modulates TNR instability in a similar manner as wild-type pol β. Our results further suggest that the individuals who carry pol βR137Q polymorphic variant, do not exhibit a higher risk of development of TNR instability and its associated diseases than individuals who have wild-type pol β.

## Materials and methods

### Materials

DNA oligonucleotide substrates were synthesized by Integrated DNA Technologies (Coralville, IA, USA). The radionucleotides [γ-^32^P] ATP (6000 mCi/mmol) and Cordycepin 5’-triphosphate 3’-[α-^32^P] (5000 mCi/mmol) were purchased from PerkinElmer Inc. (Boston, MA). T4 polynucleotide kinase, terminal deoxynucleotidyl transferase and deoxynucleoside 5’-triphosphates (dNTPs) were purchased from Thermo Fisher Scientific (Waltham, MA). Micro Bio-Spin 6 Columns were purchased from Bio-Rad Laboratories (Hercules, CA). Pierce Avidin Agarose resin was from Thermo Fisher Scientific (Waltham, MA). QuikChange II XL Site-Directed Mutagenesis kit was purchased from Agilent Technologies (Santa Clara, CA). All other standard chemical reagents were from Thermo Fisher Scientific (Waltham, MA) and Sigma-Aldrich (St. Louis, MO). Purified enzymes including APE1, pol β, FEN1 and LIG I were made according to the procedures described previously [71, 72].

### Oligonucleotide substrates

The 100 nt oligonucleotide substrates contain (CAG)_20_ repeats or (CTG)_20_ repeats with a tetrahydrofuran (THF), an analog of a modified abasic site. The THF residue substituted the first or tenth repeat unit of the (CAG)_20_ or (CTG)_20_ containing substrates. This mimics the scenario that the damage occurred at the 5’- end or in the middle of the repeat tract. Substrates were constructed by annealing the damaged strand with the template strand at a molecular ratio of 1:2. A strand of DNA fragment containing (CAG)_20_ or (CTG)_20_ repeats without damage was used as a size marker. The sequences of substrates are listed in S1 Table.

### Construction of pol βR137Q variant expression vector and expression and purification of pol βR137Q variant protein

The expression vector of pol βR137Q variant was constructed by site-directed mutagenesis using the encoding region of wild-type pol β-(His)_6_ cloned in pET15b vector as the template. Site-directed mutagenesis was conducted with the QuickChange II XL Site-Directed Mutagenesis Kit. A forward PCR primer and a reverse primer (S1 Table) were used for PCR reactions under the conditions as follows: 1 cycle of 95°C for 30 s; then 16 cycles of 95°C for 30 s, 52°C for 1 min, 68C for 7 min. The expression vector with pol βR137Q variant with a (His)_6_ tag was then transformed into *E*. *Coli* BL21DE3 (Aligent Techologies, Santa Clara, CA) for their expression according to the procedures described previously [71, 72]. Briefly, cell pellets were resuspended in the lysis buffer that contains 50 mM NaH_2_PO_4_, 30 mM NaCl, 10 mM imidazole, 10 mM ethylenediaminetetraacetic acid (EDTA), 10 mM dithiothreitol (DTT), 1 mM phenylmethylsulfonyl fluoride (PMSF), 1 mM benzamidin, 1 μg/ml leupeptin and 1 μg/ml pepstatin A. Soluble proteins and cell debris were then separated by centrifugation at 12,000 rpm, 4°C for 30 min. The supernatant was subjected to Ni-NTA agarose column from Qiagen (Hilden, Germany) for purification. Proteins were eluted by elution buffer containing 30 mM 4-(2-hydroxyethyl)-1-piperazineethane-sulfonic acid (HEPES), pH7.5, 300 mM NaCl, 500 mM imidazole, 10 mM EDTA and 10 mM DTT. Peak fractions were collected and dialyzed into buffer that contains 30 mM HEPES, pH 7.5, 0.5% inositol, 1.7M (NH_4_)_2_SO_4_ and 1 mM PMSF. Proteins were separated by phenyl sepharose 6 fast flow column (GE Healthcare Bio-Science, Uppsala, Sweden). Eluted peak fractions were combined and dialyzed into 30 mM HEPES, pH 7.5, 0.5% inositol, 30 mM KCl, 1 mM EDTA and 1 mM PMSF. Proteins were then subjected to purification with the Q sepharose (GE Healthcare Bio-Science, Uppsala, Sweden). Peak fractions were combined and dialyzed into storage buffer that contains 30 mM HEPES, 100 mM KCl. 20% glycerol and 1mM PMSF, aliquoted and frozen at -80°C for storage.

### *In vitro* reconstituted BER

Reconstituted BER was performed by incubating purified APE1, wild-type pol β or pol βR137Q variant, along with FEN1, LIG I and (CAG)_20_ or (CTG)_20_ substrates (25 nM) containing a THF residue in a reaction mixture (20 μl) that contained 5 mM MgCl_2_, 50 μM dNTPs, 2 mM ATP, 50 mM Tris-HCl (pH 7.5), 50 mM KCl, 0.1 mg/ml BSA, 0.1 mM EDTA and 0.01% NP-40. Reaction mixtures were assembled on ice and incubated at 37°C for 15 min. Reactions were terminated with 2X stopping buffer (95% formamide and 10 mM EDTA) and incubation at 95°C for 10 min. Substrates and products were then separated in 15% urea-denaturing polyacrylamide gel (PAGE) and detected by a Pharos FX Plus PhosphorImager (Bio-Rad Laboratory, CA).

### Probing of secondary structures formed in a TNR tract by S1 nuclease

The formation of hairpin structures on the damaged and template strands of the (CAG)_20_ or (CTG)_20_ substrate was probed with the S1 nuclease that makes cleavage specifically on a single-strand DNA (Promega Life Science, Madison, WI). 100 nM substrates containing a THF residue that substitutes the G at the first or tenth repeat were initially incubated with 10 nM APE1 in the absence or presence of 5 nM wild-type pol β or pol βR137Q for 30 min in the BER buffer as described previously. Subsequently, the 10 μl reaction mixtures were subjected to S1 nuclease digestion at 37°C for 1, 3, 5, 10, 15 min, respectively with 5 μl S1 nuclease reaction mixtures that contained optimized concentrations of S1 nuclease in the buffer containing 30 mM sodium acetate (pH 4.6), 50 mM NaCl, 1 mM ZnCl_2_, 0.5 mg/ml denatured calf thymus DNA and 5% glycerol. The S1 nuclease digestion was optimized by employing 25 U and 2 U of S1 nuclease for the digestion of the the damaged strand and template strand of the (CAG)_20_ containing substrate that contains a THF located at the first repeat, respectively; and by employing 3 U, 25 U and 5 U of S1 nuclease for the digestion of the upstream damaged strand, downstream damaged strand and the template strand of the (CAG)_20_ containing substrate that contains a THF located at the tenth repeat, respectively. Reactions were then quenched with addition of 2 μg proteinase K and incubation at 55°C for 30 min. Reaction mixtures were then mixed with the same volume of 2X stopping buffer and denatured at 95°C for 10 min. Substrates and products were separated in an 18% urea-denaturing PAGE and detected by a PhosphorImager.

### Enzymatic activity assay

Pol β DNA synthesis activity were determined by incubating wild-type pol β or pol βR137Q variant in the absence or presence of PCNA with 25 nM (CAG)_20_ or (CTG)_20_ substrate with a THF residue in reaction buffer containing 50 mM Tris-HCl, pH 7.5, 50 mM KCl, 0.1 mM EDTA, 0.1 mg/ml bovine serum albumin and 0.01% Nonidet P-40 along with 50 μM dNTPs and 5 mM Mg^2+^ at 37°C for 15 min. FEN1 cleavage activity on the substrates was measured in the absence and presence of wild-type pol β or pol βR137Q variant at 37°C for 15 min in reaction buffer under the conditions described previously. The 20 μl of reaction mixture was assembled on ice, and reaction was quenched by addition of 20 μl stopping buffer and incubation at 95°C for 10 min. Substrates and products were separated by a 15% urea denaturing gel and detected by a PhosphoImager.

### Sizing analysis of BER products by DNA fragment analysis

To isolate a repaired strand specifically, the template strand of all substrates was tagged by a biotin residue at the 5’-end. BER reactions were terminated with 1 μl of 100 mM EDTA, and reaction mixtures were incubated with 50 μl avidin agarose beads (Pierce-Thermo Scientific, Rockford, IL) for 2 hrs, allowing the binding of avidin beads to the biotin on the template strand. Reaction mixtures were then subjected to incubation with 0.15 M NaOH at room temperature for 15 min with rotation allowing separation of repaired strands from the template strands. This was followed by 2 min centrifugation at 5000 rpm pelleting the template strands bound by avidin beads. The repaired strands in the supernatant were precipitated with ethanol and subsequently dissolved in TE buffer for PCR amplification and size analysis. Repaired products were amplified through PCR with the AmpliTaq Gold 360 DNA polymerase Kit (Applied Biosystems, Foster City, CA) at the conditions: denaturation at 95°C for 30 s, annealing at 50°C for 30 s, extension at 72°C for 90 s for 35 cycles with a final extension at 72°C for 1 hr. CAG forward primer and 6-carboxyfluorescein (6-FAM) tagged CAG reverse primer, were used for PCR amplification of the repaired products from the (CAG)_20_ substrate with a THF at 5’-end or in the middle of the repeat tract. CTG-1 forward primer and 6-FAM tagged reverse primer were used for PCR amplification of the repaired products from the (CTG)_20_ substrate with a THF at 5’-end of the repeat tract; while CTG-10 forward primer and 6-FAM tagged reverse primer were used to amplify the repaired products of (CTG)_20_ substrate with a THF in the middle of the repeat tract. The sequences of the primers are indicated in S1 Table. PCR products along with the size marker MapMarker 1000 (Bioventures, Murfreesboro, TN) were then subject to capillary electrophoresis via an ABI 3130XL Genetic Analyzer (Applied Biosystems, Foster City, CA) at Florida International University DNA sequencing core facility. The sizes of PCR products were determined by DNA fragment analysis with the Peak Scanner version 1.0 software (Applied Biosystems, Foster City, CA).

## Results

### Pol βR137Q polymorphic variant exhibits weaker DNA synthesis activity than wild-type pol β in the context of CAG and CTG repeats

**Fig 1:**
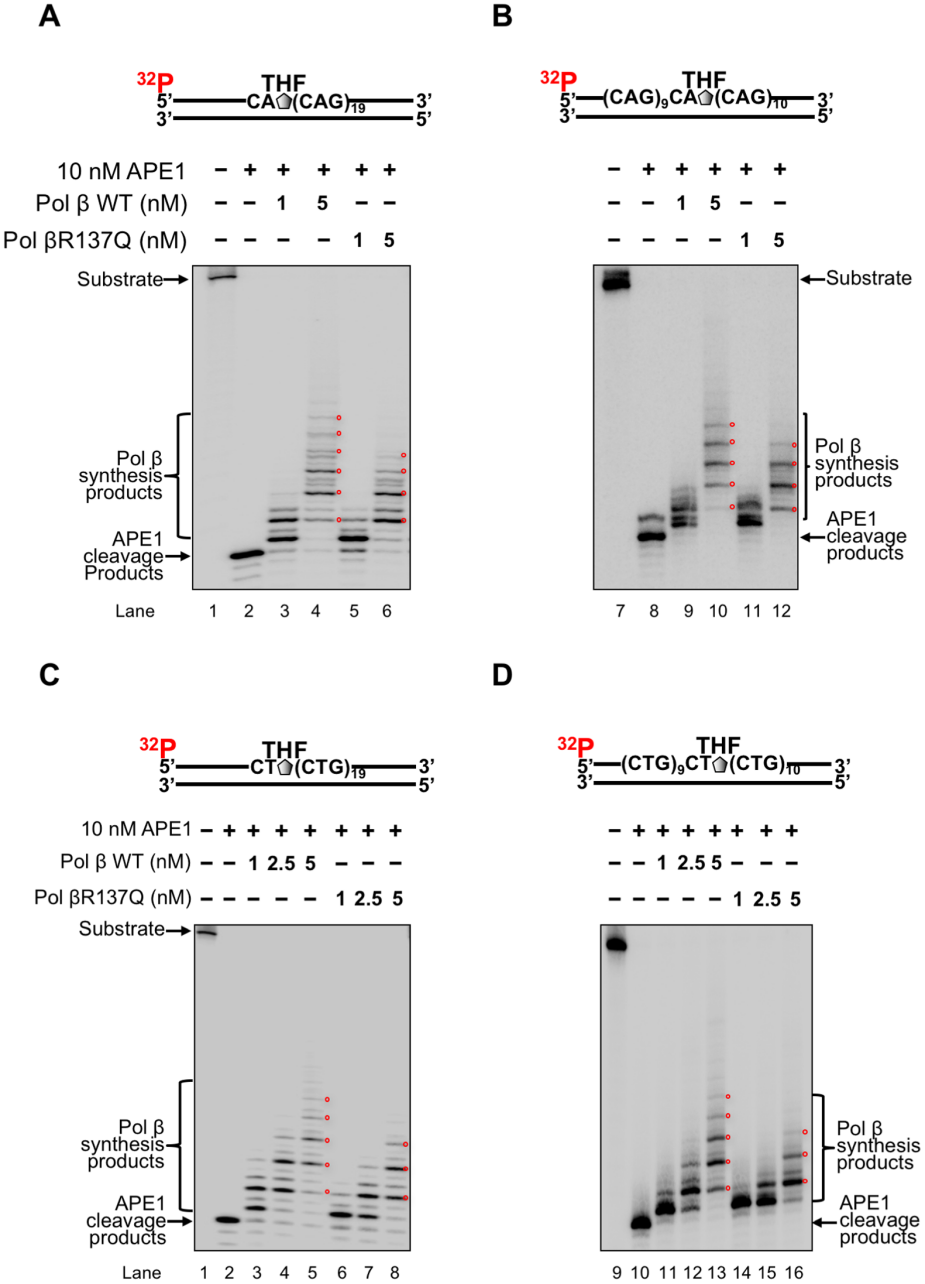
Pol βR137Q variants exhibits weak DNA synthesis activity during BER of a base lesion in context with (CAG)_20_ and (CTG)_20_ repeats. DNA synthesis activity of wild-type pol β (pol β WT) or pol βR137Q variant at various concentrations, was measured with 25 nM substrates containing (CAG)_20_ and (CTG)_20_ with a THF residue. DNA synthesis activity of pol β on the (CAG)_20_ and (CTG)_20_ repeat substrates was measured at the concentrations of 1 nM, 2.5 nM and 5 nM. Substrates were ^32^P-labeled at the 5’-end of the damaged strand. **(A)** and **(B)** Measurement of DNA synthesis activity with (CAG)_20_ substrate containing a THF at the first and tenth repeat, respectively. Lanes 1 and 11 represent substrate only. Lane 2 and 12 represent APE1 cleavage products. Lanes 3–4 and 13–14 represent pol β WT synthesized products. Lanes 5–6 and 15–16 represent pol β R137Q variant synthesized products. **(C)** and (**D**) Measurement of DNA synthesis activity assay in the context with (CTG)_20_ substrate with a THF at the first and tenth repeat, respectively. Lanes 1 and 15 represent substrate only. Lane 2 and 16 represent the reaction with APE1 along. Lanes 3–5 and 17–19 illustrate the reactions with pol β WT. Lanes 6–8 and 20–22 represent reactions with pol βR137Q. The red circles superimposed in the gels indicate the locations of the synthesized products of pol β WT or pol βR137Q.

Since pol β DNA synthesis plays a crucial role in mediating TNR expansion [43] and deletion during BER [45] in a damage location dependent manner [44], we initially examined the DNA synthesis activity of pol βR137Q with the (CAG)_20_ or (CTG)_20_ repeat substrate containing an abasic site (THF) located at the 5'-end or in the middle of the repeat tract. We found that with the damage located at the 5’-end of the (CAG)_20_ repeat tract, pol βR137Q and wild-type pol β at 1 nM, mainly inserted 1 and 3 nucleotides (Fig 1A, compare lane 5 with lane 3). However, 5 nM pol βR137Q variant inserted up to 4 repeats (Fig 1A, lane 6), whereas the same concentration of wild-type pol β inserted up to 6 repeats (Fig 1A, lane 4). With the damage located in the middle of the repeat tract, pol βR137Q inserted up to 3 nucleotides and wild-type pol β inserted up to 5 nucleotides (Fig 1B, compare lane 11 with lane 9). However, 5 nM pol βR137Q variant inserted up to 4 repeats, whereas the same concentration of wild-type pol β inserted up to 5 repeats (Fig 1B, compare lane 12 with lane 10). Similarly, for the (CTG)_20_ substrate with the damage at the 5'-end, 1 nM pol βR137Q mainly inserted 1 nucleotide, while the same concentration of wild-type pol β mainly inserted 3 nucleotide on the substrate (Fig 1C, compare lane 6 and lane 3). 2.5 nM and 5 nM pol βR137Q inserted up to 2 and 3 repeats with the same substrate while wild-type pol β inserted up to 3 or 5 repeats (Fig 1C, compare lanes 7 and 8 with lanes 4 and 5). With the damage located in the middle of the (CTG)_20_ substrate, 1 nM and 2.5 nM pol βR137Q variant inserted 2 nucleotides and 1 repeat, respectively (Fig 1D, lanes 14–15), whereas the wild-type pol β at the same concentrations inserted 1 nucleotide and up to 2 repeats, respectively (Fig 1D, lanes 11–12). At the concentration of 5 nM, pol βR137Q variant inserted up to 3 repeats, while wild-type pol β inserted up to 5 repeats (Fig 1D, compare lane 16 with lane 13). The results indicated that pol βR137Q exhibited weaker DNA synthesis activity than wild-type pol β in the context of TNRs.

To compare the gap-filling synthesis activity of pol βR137Q variant and wild-type pol β at low concentrations, the gap-filling synthesis of pol βR137Q variant and wild-type pol β at the concentrations of 0.1 nM, 0.2 nM and 0.5 nM was measured with the substrates containing the (CAG)_20_ and (CTG)_20_ with a THF at the 5’-end or in the middle of the repeat tract. For the (CAG)_20_ and (CTG)_20_ substrates with a THF at the 5’-end, both wild-type pol β and pol βR137Q incorporated 1 nucleotide at the concentrations of 0.1 nM and 0.2 nM (S1A and S1C Fig, lanes 3–4 and 6–7). Moreover, wild-type pol β and pol βR137Q variant at 0.5 nM incorporated up to 3 nucleotides (S1A and S1C Fig, lanes 5 and 8). Similarly, for the (CAG)_20_ and (CTG)_20_ substrates with a THF in the middle, wild-type pol β and pol βR137Q variant at the concentrations of 0.1 nM, 0.2 nM and 0.5 nM mainly inserted one nucleotide (S1B and S1D Fig, lanes 11–16). The results indicated that pol βR137Q variant exhibited similar gap-filling synthesis activity as wild-type pol β at low concentrations. We also examined the gap-filling synthesis activity of wild-type pol β and pol βR137Q variant at low concentrations of 0.1 nM, 0.2 nM and 0.5 nM on the 1 nt-gap substrates with (CAG)_20_ repeats or a random DNA sequence. For the 1-nt gap substrate with (CAG)_20_ repeats, both wild-type pol β and pol βR137Q variant mainly inserted one nucleotide to fill in the gap at all tested concentrations (S1E Fig, lanes 2–7). For the substrate containing a random DNA sequence, both wild-type pol β and pol βR137Q inserted one nucleotide to fill in the gap at concentrations of 0.1 nM and 0.2 nM (S1F Fig, lanes 9–10 and 12–13). Furthermore, both wild-type pol β and pol βR137Q at 0.5 nM inserted additional 2 nucleotides to displace the downstream flap (S1F Fig, lanes 11 and 14). The results indicated that pol βR137Q variant at low concentrations exhibited slightly weaker one-nucleotide gap-filling synthesis compared to that of wild-type pol β on the 1-nt gap substrate with (CAG)_20_ repeats (S1E Fig) and similar gap filling synthesis and strand displacement synthesis activity with the wild-type polymerase on the 1-nt gap substrates with a random DNA sequence (S1F Fig).

### PCNA does not affect DNA synthesis activity of pol βR137Q variant

**Fig 2:**
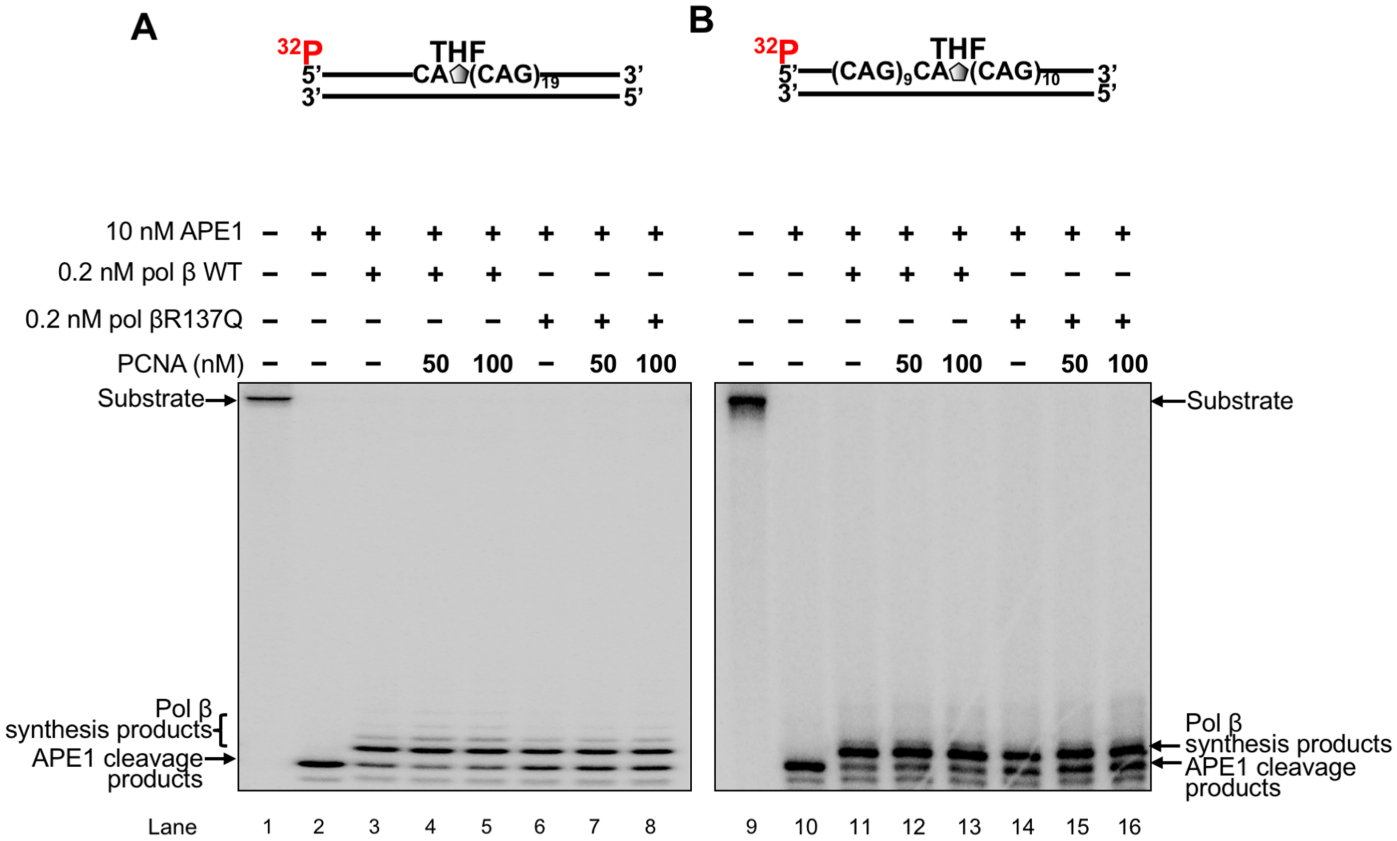
PCNA does not affect DNA synthesis activity of pol β WT and pol βR137Q during BER of a base lesion on (CAG)_20_ repeats. The effects of PCNA on pol β was examined by determining pol β DNA synthesis in the presence of 0.2 nM of pol β WT or pol βR137Q along with 50 nM and 100 nM PCNA. 25 nM substrates were ^32^P-labeled at the 5’-end of the damaged strand of the substrates. **(A)** and **(B)** Pol β DNA synthesis in the presence of PCNA with the (CAG)_20_ substrate containing a THF located at the first repeat and tenth repeat, respectively. Lanes 1 and 9 represent substrate only. Lanes 2 and 10 represent reaction with APE1. Lanes 3 and 11 illustrate reactions with pol β WT. Lanes 6 and 14 represent reactions with pol βR137Q. Lanes 4–5 and 12–13 represent reactions with pol β WT in the presence of increasing concentrations of PCNA. Lanes 7–8 and 15–16 represent reaction mixtures with pol βR137Q in the presence of increasing concentrations of PCNA.

As a BER cofactor, PCNA can stimulate the activities of both FEN1 and LIG I during long patch BER [73, 74]. However, it remains unknown whether PCNA may affect pol β DNA synthesis activity although it has been found that PCNA can physically interact with pol β [75]. Since pol βR137Q variant has weak interaction with PCNA [64], it is possible that this can affect pol βR137Q DNA synthesis activity, promoting TNR instability. To test this, high concentrations of PCNA (50 nM and 100 nM) were employed to determine if PCNA could stimulate pol β DNA synthesis activity in the context of TNRs at a low concentration (0.2 nM) of wild-type pol β or pol βR137Q variant. We found that in the absence of PCNA or presence of 50 nM and 100 nM PCNA, at 0.2 nM, both pol βR137Q and wild-type pol β generated a similar amount of one nucleotide insertion product during BER of an abasic lesion (THF) located at either the 5’-end (Fig 2A, compare lanes 4–5 with lane 3 and lanes 7–8 with lane 6) or in the middle of the (CAG)_20_ repeat substrates (Fig 2B, compare lanes12-13 with lane 11 and lanes 15–16 with lane 14). The results showed that at the high concentration of PCNA, no stimulatory effect was detected for the gap-filling synthesis activity of wild-type pol β and pol βR137Q variant (Fig 2), indicating that PCNA did not affect the processivity of pol β WT and pol βR137Q variant either. This further demonstrates that the impaired interaction between PCNA and pol βR137Q variant did not affect the polymerase gap-filling synthesis activity of the pol β variant.

### The weak DNA synthesis of pol βR137Q variant leads to weak FEN1 cleavage during BER in a TNR tract

**Fig 3:**
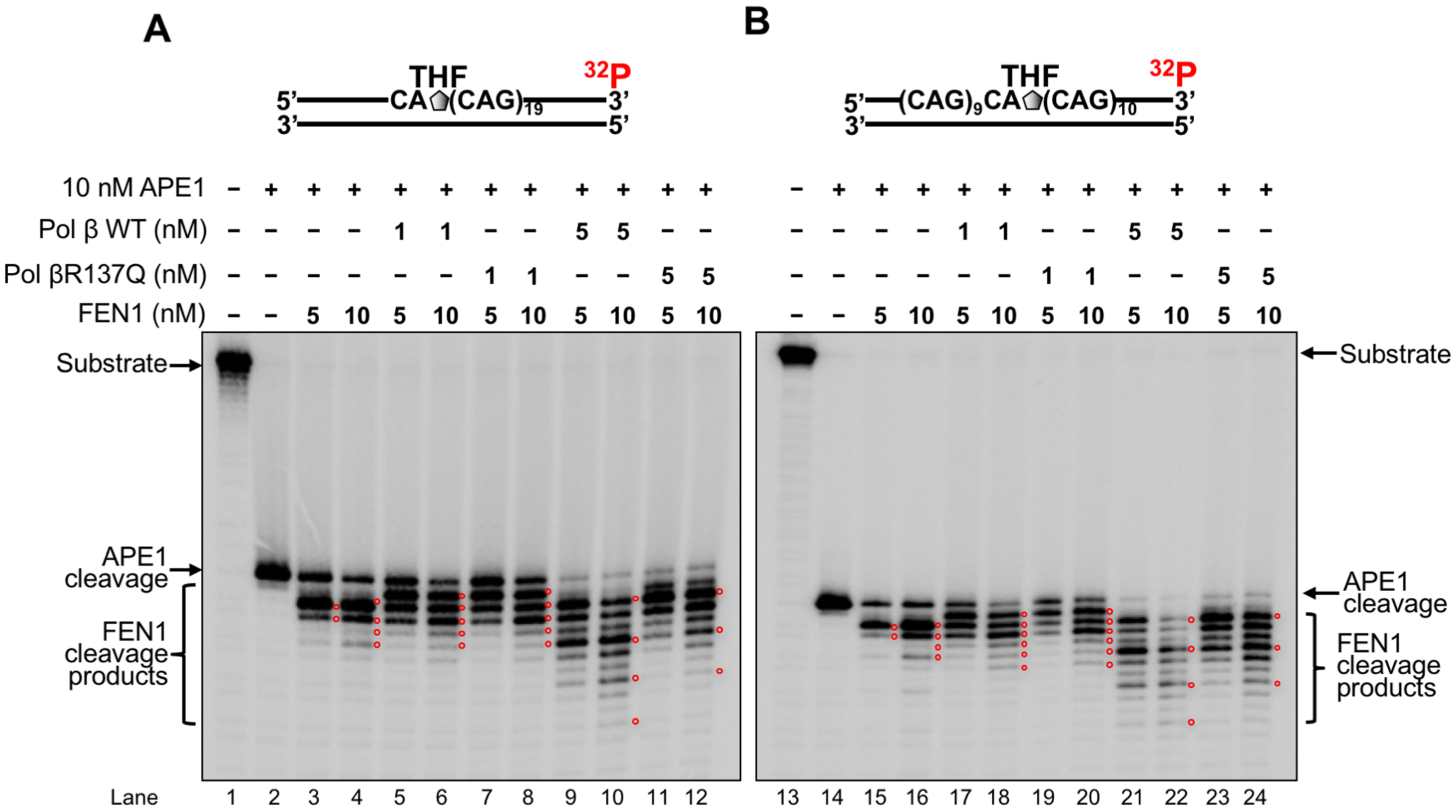
Pol βR137Q leads to weak FEN1 cleavage during BER in the context of (CAG)_20_ repeats. FEN1 flap cleavage activity at 5 nM and 10 nM on the (CAG)_20_ substrate with a THF located at the first repeat (**A**) or tenth repeat (**B**) was measured in the presence of 1 nM and 5 nM pol β. Lanes 1 and 13 represent the substrate only. Lanes 2 and 14 represent reactions with APE1 alone. Lanes 3–4 and lanes 15–16 represent reactions with FEN1 alone. Lanes 5–6 and 17–18 represent reactions with FEN1 in the presence of 1 nM pol β WT. Lanes 7–8 and 19–20 illustrate reactions with FEN1 in the presence of 1 nM pol βR137Q. Lanes 9–10 and 21–22 represent reactions with FEN1 in the presence of 5 nM pol β WT. Lanes 11–12 and 23–24 illustrate reactions with FEN1 in the presence of 5 nM of pol βR137Q variant. Substrates were ^32^P-labeled at the 3’-end of the damaged strand. The red circles superimposed in the gels indicate the locations of FEN1 cleavage products.

Since pol β DNA synthesis in the context of TNR tracts facilitates FEN1 cleavage by creating a TNR flap during BER [40, 44, 45], we then examined the effect of pol βR137Q on FEN1 cleavage activity by measuring FEN1 activity in the presence of 1 nM or 5 nM polβR137Q variant during repair of an abasic site located at the 5’-end and in the middle of the (CAG)_20_ repeat substrates (Fig 3). We found that in the absence of pol βR137Q variant or wild-type pol β, FEN1 cleaved up to 3 nucleotides at 5 nM and up to 5 nucleotides at 10 nM on the substrate with the lesion at the 5’-end (Fig 3A, lanes 3–4). However, FEN1 still removed up to 3 nucleotides at 5 nM and up to 5 nucleotides at 10 nM in the presence of 1 nM pol βR137Q variant or wild-type pol β (Fig 3A, compare lanes 5–8 with lanes 3–4). In the presence of 5 nM pol βR137Q, the same concentrations of FEN1 (5 nM and 10 nM) removed up to 2 and 3 CAG repeats from the CAG repeat substrate (Fig 3A, lanes 11–12), whereas in the presence of 5 nM wild-type pol β, FEN1 at 5 nM and 10 nM of removed up to 3 and 4 repeats, respectively (Fig 3A, lanes 9–10). Similarly, FEN1 cleavage activity on the substrate with the lesion in the middle of the repeat tract, was not altered significantly in the presence of 1 nM pol βR137Q and wild-type pol β by comparing to in the absence of pol βR137Q and wild-type pol β, FEN 1 cleaved up to 3 and 5 nucleotides at 5 nM and 10 nM in the absence of pol β, respectively (Fig 3B, lanes 14–15); with additional 1 nucleotide cleaved by FEN1 in the presence of 1 nM pol βR137Q variant and wild-type pol β at the concentration of 10 nM of FEN1 (Fig 3B, compare lane 18 and lane 20 with lane 16). However, in the presence of 5 nM pol βR137Q, FEN1 (5 nM and 10 nM) mainly cleaved 2 and 3 CAG repeats, respectively, compared to 3 and 4 CAG repeats, respectively, removed by FEN1 (5 nM and 10 nM) in the presence of 5 nM wild-type pol β (Fig 3B, compare lanes 23–24 with lanes 21–22). The results indicate that the weak DNA synthesis of pol βR137Q resulted in weaker FEN1 cleavage of TNRs than that from wild-type pol β by creating a shorter repeat flap.

### Pol βR137Q variant leads to small TNR deletions during BER of an abasic site in a TNR tract

**Fig 4:**
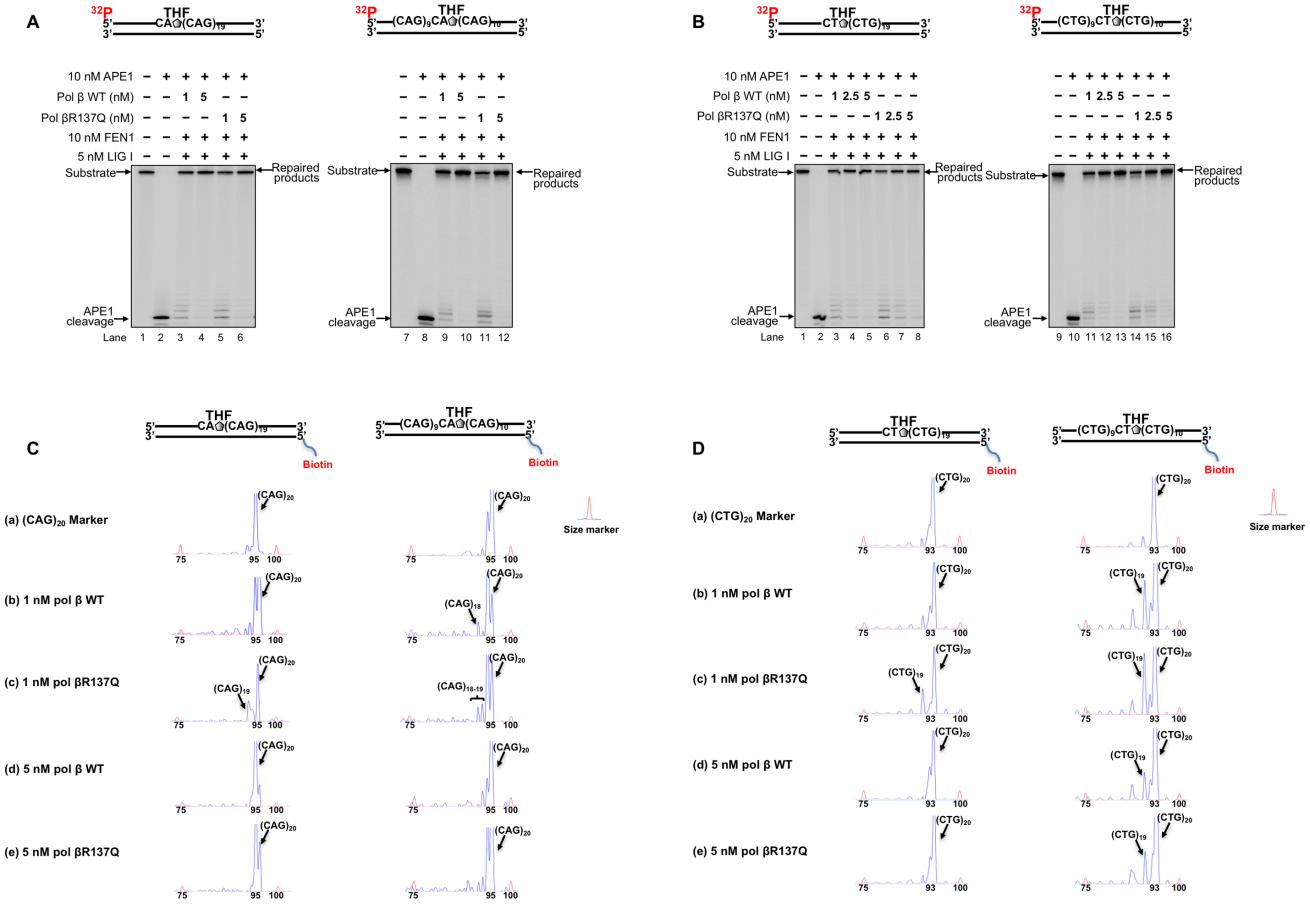
Effects of pol βR137Q on the instability of (CAG)_20_ and (CTG)_20_ repeats. BER was reconstituted with 10 nM of APE1, 10 nM FEN1, 5 nM LIG I and increasing concentrations of pol β WT or pol βR137Q (1 nM and 5 nM on (CAG)_20_ substrate and 1 nM, 2.5 nM and 5 nM on (CTG)_20_ substrate) along with 25 nM substrates. **(A)** BER reconstituted with the (CAG)_20_ substrate with a THF located at the first repeat (left panel) or the tenth repeat (right panel). Lane 1 and lane 7 represent substrates only. Lane 2 and 8 represent reactions with APE1 only. Lanes 3–4 and 9–10 represent reactions with pol β WT. Lanes 5–6 and 11–12 represent reactions in the presence of pol βR137Q. **(B)** BER reconstituted with the (CTG)_20_ substrate with a THF located at the first repeat (left panel) and the tenth repeat (right panel). Lane 1 and lane 9 represent substrates only. Lane 2 and 10 represent reactions with APE1 only. Lanes 3–5 and 11–13 present reactions with pol β WT. Lanes 6–8 and 14–16 illustrate reactions with pol βR137Q. Repaired products were subjected to PCR and capillary electrophoresis and analyzed by DNA fragment analysis to determine the length. **(C)** DNA fragment analysis of the repaired products of from the substrate with a lesion located at the first (left panel) and the tenth of the (CAG)_20_ substrate. **(D)** DNA fragment analysis of the repaired products of from the substrate with a lesion located at the first (left panel) and the tenth of the (CTG)_20_ substrate. Panel a represent a (CAG)_20_ or (CTG)_20_ marker without damage. Panel b and c indicate the sizes of the repaired products in the presence of 1 nM pol β WT or 1 nM R137Q, respectively. Panel d and e represent the sizes of the repaired products in the presence of 5 nM pol β WT or 5 nM pol βR137Q, respectively.

To further determine whether the weak DNA synthesis of pol βR137Q variant may affect trinucleotide repeat instability, we reconstituted BER of an abasic lesion in the context of a TNR tract with the pol β variant and the (CAG)_20_ or (CTG)_20_ substrates containing the lesion at the 5'-end or in the middle of the repeat tract and measured the length change of the repeats (Fig 4). We found that 1 nM and 5 nM wild-type pol β and pol βR137Q led to repaired products during BER of an abasic lesion in the context of a (CAG)_20_ (Fig 4A, lanes 3–4 and lanes 9–10 for wild-type pol β; Fig 4A lanes 5–6 and lanes 11–12 for pol βR137Q) or (CTG)_20_ repeat tract (Fig 4B, lanes3, 5,11 and 13 for wild-type pol β; Fig 4B lanes 6, 8, 14 and 16 for pol βR137Q). Further analysis on the repeat size of the repaired products showed that BER reconstituted with 1 nM pol βR137Q or wild-type pol β resulted in a product with one repeat deletion with the CAG and CTG substrates containing the base lesion at the 5’-end (Fig 4C and 4D, left panel, panel b and c), whereas 5 nM pol βR137Q or wild-type pol β led to full length repaired products (Fig 4C and 4D, left panel, panel d and e). BER reconstituted with 1 nM pol βR137Q or wild-type pol β and the substrates with the lesion in the middle, resulted in the repaired products with one or two repeat deletion (Fig 4C and 4D, right panel, panel b and c). 5 nM pol βR137Q and wild-type enzyme decreased the amount of the deletion product (Fig 4C and 4D, right panel, panel d and e). The results indicate that pol βR137Q exhibited the same ability as wild-type pol β enzyme to modulate TNR instability by causing one or two repeat deletions.

### Pol βR137Q variant skips over a small loop on the template strand of a TNR repeat tract

**Fig 5:**
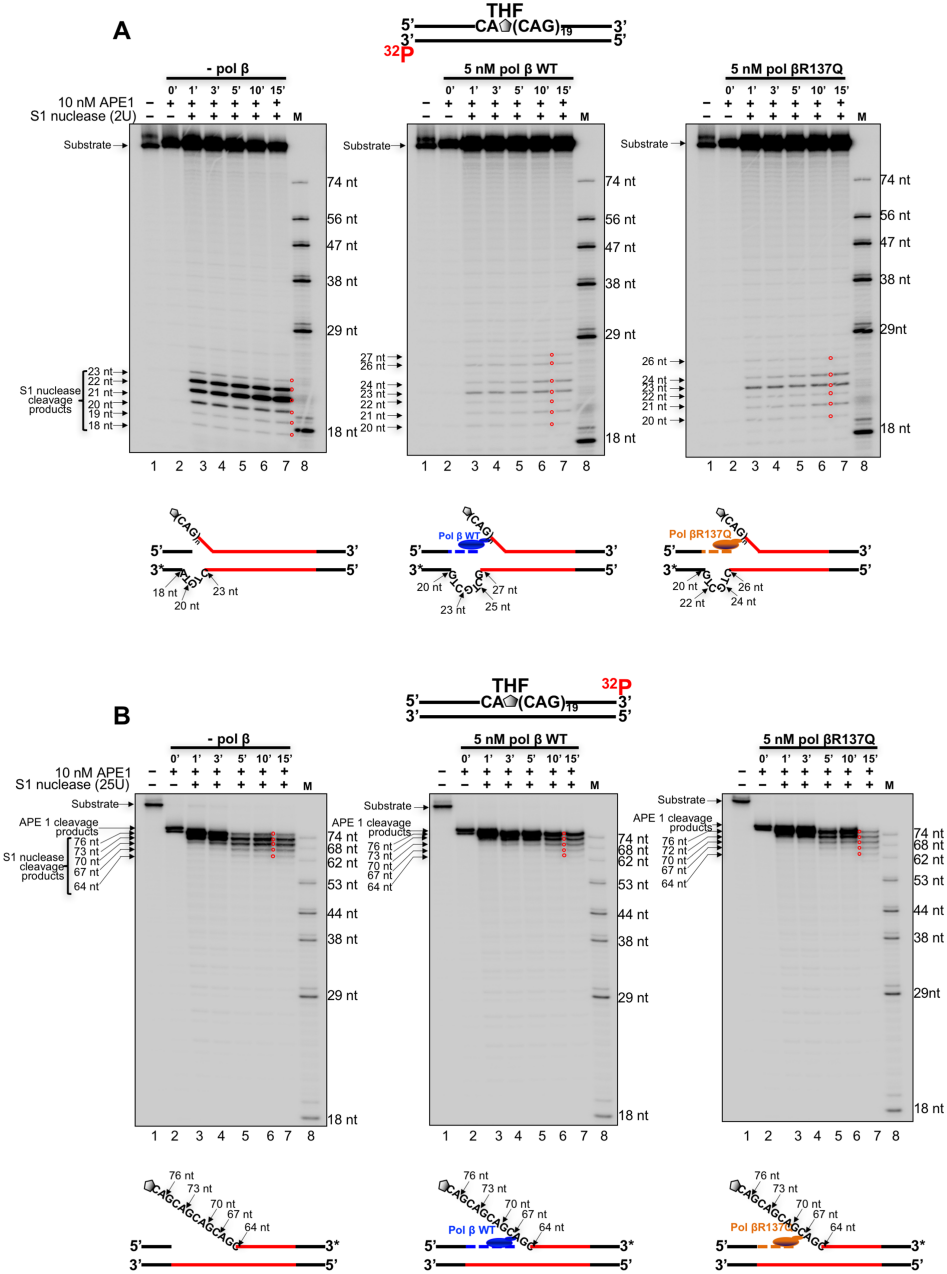
S1 nuclease probing of loops formed in (CAG)_20_ repeats with a THF located at the first CAG of the (CAG)_20_ repeat substrate in the absence and presence of pol β WT or pol βR137Q DNA synthesis. The S1 nuclease probing of a loop located at the template strand (**A**) and the downstream damage strand of the (CAG)_20_ substrate. (**B**) Both the template strand and damage strand were labeled at the 3’-end. Substrates were incubated with 2 U or 25 U S1 nuclease. Panels on the left, in the middle and on the right, correspond to S1 nuclease cleavage results in the presence of APE1 alone, pol β WT and pol βR137Q, respectively. Lane 1 represents the substrate only. Lane 2 represents the reaction with APE1 alone. Lanes 3–7 represent reaction mixtures with S1 nuclease in the absence or the presence of pol β DNA synthesis at different time intervals. Lane 8 represents a synthesized marker with different sizes of DNA fragments. The schemes below the gels illustrate S1 nuclease cleavage patterns in the absence and presence of pol β WT and pol βR137Q. The red circles superimposed in the gels indicate the locations of the S1 nuclease digestion products.

Our previous studies have shown that secondary structures such as hairpins and loops can form readily during BER in a TNR tract [43–45], and pol β can readily skip over the structures on the template strand [45]. The “skip over” refers to a scenario where the polymerase encountered the secondary structures such as a hairpin or loop structure formed on the template strand, it performed DNA synthesis to bypass the secondary structures other than copying through the inside of the secondary structures. We have further demonstrated that pol β DNA synthesis modulates TNR expansion and deletion by altering the balance between the synthesis of TNRs and removal of the repeats by FEN1 flap cleavage [44, 45]. Since the weak DNA synthesis activity of pol βR137Q resulted in weak FEN1 cleavage activity, it is possible that the pol β polymorphic variant can alter TNR instability. To test this possibility, a single-stranded DNA specific endonuclease Aspergillus S1 nuclease [76] was used to probe the secondary structures that formed on the template strand of the (CAG)_20_ repeat substrate in the absence or presence of DNA synthesis of pol β at the time intervals of 1 min-15 min. We found that in the absence of pol β, S1 cleavage on the template strand of the (CAG)_20_ substrate with a THF located at the 5’-end, resulted in the products of 18 nt, 19 nt, 20 nt, 21 nt, 22 nt and 23 nt (Fig 5A, left panel, lanes 3–7). This indicated the formation of a small loop containing one CTG repeat adjacent to the 3’-end of the flanking region of the repeat tract (Fig 5A, the scheme below the gel). In the presence of DNA synthesis of wild-type pol β and pol βR137Q variant, S1 nuclease cleavage led to the products of 21 nt, 23 nt, 24 nt, 26 nt and 27 nt indicating the formation of a loop containing two CTG repeats (Fig 5A, the panels in the middle and on the right, lanes 3–7). The results indicated that in both the absence and presence of DNA synthesis of both pol βR137Q variant and wild-type pol β, a small (CTG)_2_ loop formed on the template strand. This further indicated that the DNA synthesis of pol βR137Q variant and wild-type pol β only altered the position of the loop by shifting the loop toward the 5’-end of the template. We also observed that in the presence of DNA synthesis of wild-type pol β and pol βR137Q variant, the S1 nuclease cleavage products were much weaker than those generated in the absence of pol β DNA synthesis. This was because pol β DNA synthesis copied through the template strand, which converted the S1 nuclease-sensitive ssDNA loop region on the template strand to a S1 nuclease-resistance dsDNA region. Furthermore, the pol β DNA synthesis displaced the downstream damaged strand, exposing the annealed template strand as a ssDNA region. This then allowed the ssDNA region on the template strand approximately 1 repeat to shift towards the 5’-end. This is consistent with the 1 repeat insertion observed in DNA synthesis (Fig 1A). Further analysis of S1 nuclease cleavage on the downstream strand of the damaged strand of the substrate showed that S1 nuclease resulted in the products of 64 nt, 67 nt, 70 nt, 73 nt and 76 nt in the absence and presence of both pol βR137Q and wild-type pol β indicating that the downstream strand formed a single-stranded (CAG)_5_ flap (Fig 5B, lanes 3–7, and schemes below the gels), and DNA synthesis pol βR137Q variant and wild-type pol β did not alter the formation of the downstream flap.

**Fig 6:**
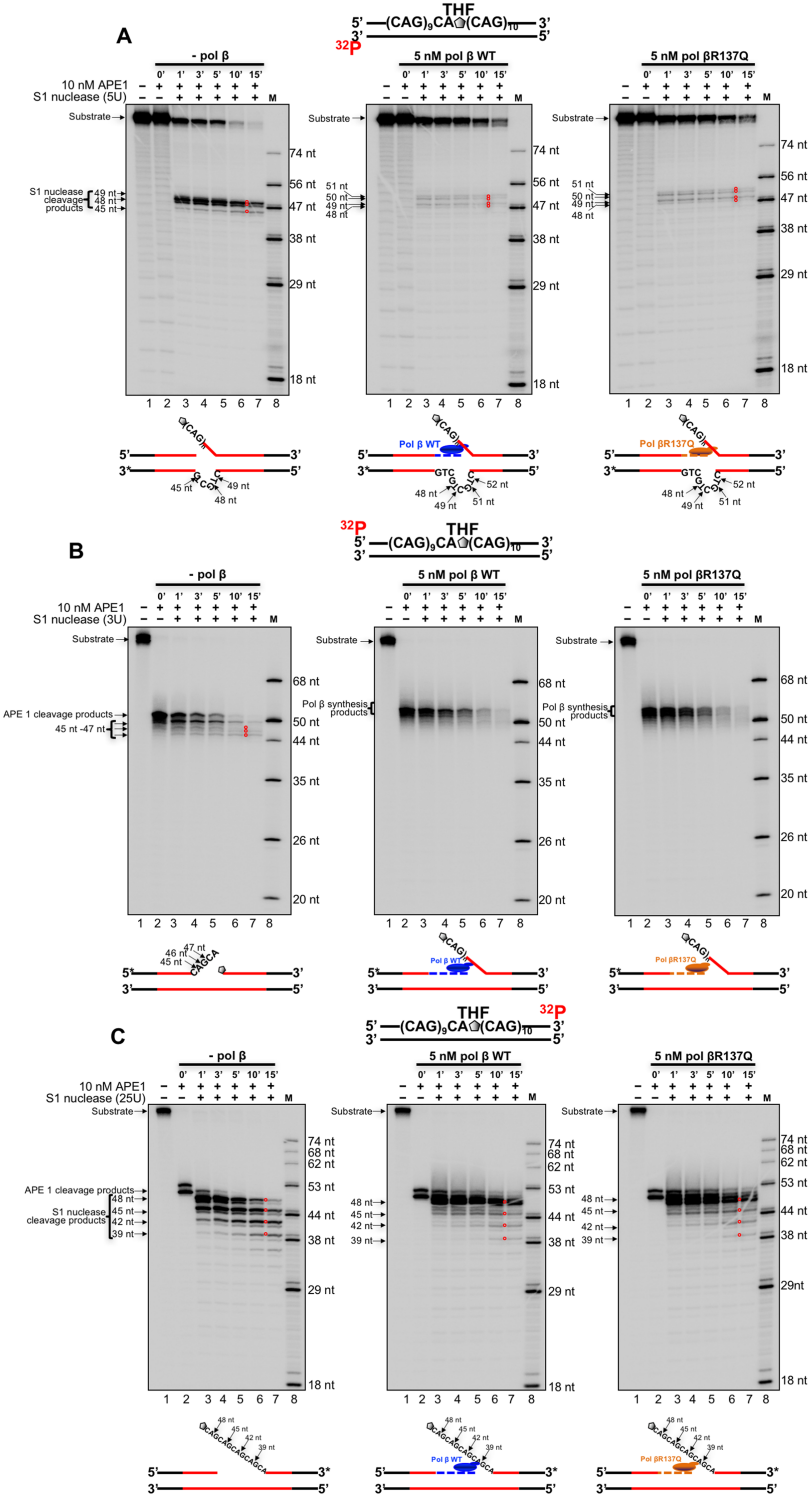
S1 nuclease probing of loops formed in (CAG)_20_ repeats with a THF located at the tenth CAG of the (CAG)_20_ repeat substrate in the absence and presence of pol β WT or pol βR137Q DNA synthesis. S1 nuclease probing of a loop on the template strand (**A**), the upstream damaged strand (**B**) and the downstream damaged strand (**C**) of the (CAG)_20_ substrate with a THF at the tenth repeat were conducted. The template strand and downstream strand of the damaged strand were labeled at the 3’-end, and the upstream damaged strand was labeled at the 5’-end. Substrates were incubated with 5 U, 3 U and 25 U S1 nuclease in the absence and presence of pol β WT and pol βR137Q. Panels on the left, in the middle and on the right, correspond to reactions with S1 nuclease and APE1 alone, in the presence of pol β WT and in the presence of pol βR137Q, respectively. Lane 1 represents the substrate only. Lane 2 represents the reaction with APE1 alone. Lanes 3–7 represent reaction mixtures with S1 nuclease and APE1 at different time intervals. Lane 8 represents synthesized markers. The schemes below the gels illustrate S1 nuclease cleavage pattern on the different strands of the substrate. The red circles superimposed in the gels indicate the S1 nuclease digestion products.

For the (CAG)_20_ substrate with a THF in the middle of the repeat tract, S1 cleavage on the template resulted in the products of 45 nt, 48 nt and 49 nt at the time interval of 1–15 min in the absence of pol β (Fig 6A, left panel, lanes 3–7) indicating the formation of a (CTG)_2_ loop (Fig 6A, left panel, the scheme below the gel). In the presence of DNA synthesis of wild-type pol β or pol βR137Q variant, S1 nuclease cleavage on the template strand resulted in the products of 48 nt, 49 nt, 50 nt and 51 nt. This also indicated the formation of a (CTG)_2_ loop which was shifted toward the 5'-end of the template strand (Fig 6A, the panels in the middle and on the right, lanes 3–7). The size of the loop is consistent with deletion of one or two repeats resulting from BER reconstituted by wild-type pol β and pol βR137Q with the substrate (Fig 4C and 4D, the left panel). This further suggested that pol β skipped over the loop structure and performed DNA synthesis to displace the downstream strand generating a flap. To test this, we then examined S1 nuclease cleavage on the upstream strand of the damage strand of the (CAG)_20_ substrate (Fig 6B). We found that in the absence of pol β, S1 nuclease cleavage resulted in the products of 47 nt, 46 nt and 45 nt that are shorter than the APE1 cleavage product of 50 nt (Fig 6B, left panel, lanes 3–7), indicating the formation of an upstream flap containing two CAG repeats (Fig 6B, the scheme below the left panel). In the presence of S1 nuclease and DNA synthesis of wild-type pol β or pol βR137Q, a product of 53 nt, which is longer than the APE1 cleavage product of 50 nt, was detected (Fig 6B, the panels in the middle and on the right, lanes 3–7), indicating that both wild-type pol β and pol βR137Q efficiently pushed the upstream flap to re-anneal to the template strand and extended the upstream strand (Fig 6B, the schemes below the panels in the middle and on the right). This further indicated that pol β DNA synthesis displaced the downstream strand and created a flap. This can be confirmed by the FEN1 cleavage results showing that FEN1 cleaved more nucleotides in the presence of wild-type pol β or pol βR137Q variant than the ones in the absence of pol β (Fig 3B, compare lanes 18, 20, 22 and 24 to lane 16). The cleavage of the downstream strand of the substrate in the absence and presence of wild-type pol β or pol βR137Q variant resulted in the product of 39 nt, 42 nt, 45 nt and 48 nt (Fig 6C, left, middle and right panels, lanes 3–7). However, S1 nuclease cleaved more on the substrate in the absence of pol β and fewer on the substrate in the presence of pol β (Fig 6C, compare lanes 3–7 from the panels in the middle and on the right with lanes 3–7 in the panel on the left). This suggests that pol β bound to the strand break intermediate generated by APE1 5'-incision of the THF, which protected the 5’-flap downstream ssDNA from S1 nuclease cleavage. The results indicate that the weak DNA synthesis activity of pol βR137Q still exhibited the similar ability as wild-type pol β in skipping-over of a small loop as well as in performing strand-displacement synthesis to create a downstream flap during BER in the context of CAG repeats.

In order to exclude the possibility that the digestion bands may be the result of the “breathing effect”, we performed the control experiments with the nick substrates digested by S1 nuclease, where the nicks were located after the first CAG or after the tenth CAG of the (CAG)_20_ substrates. Under the same experimental condition with Figs 5A and 6A, no S1 digestion products that indicate the S1 cleavage at the site opposite the nick on the template strand were detected (S2A and S2B Fig, lanes 2–7). This indicates that S1 cleavage did not result from the “breathing effect”. This further suggests that S1 nuclease cleavage products were the result of the skipping over of the template loop by the polymerases (Figs 5A and 6A, the panels in the middle and on the right, lanes 3–7).

## Discussion

Recent studies have shown that the polymorphism of several DNA repair proteins is associated with TNR diseases and the age of disease onset. This includes the polymorphisms in OGG1(Rs1052133) (Ser326Cys) [77, 78], MSH3 (Rs26279) [56], XPC [78] and ERCC6 (rs2228528) [60]. This suggests that the polymorphisms of proteins of BER, mismatch repair (MMR) and nucleotide excision repair (NER) pathways can modulate TNR instability. In this study, for the first time, we showed that pol βR137Q exhibited weaker DNA synthesis activity than wild-type pol β in the context of TNRs (Fig 1). However, the DNA synthesis activity of pol βR137Q was not affected by PCNA during BER (Fig 2) although it is reported that this pol β variant has lost the interaction with PCNA [64]. The weak DNA synthesis of the pol β polymorphic variant further led to weak FEN1 cleavage of TNR flaps (Fig 3). Yet, we found that pol βR137Q exhibited the similar ability to that of wild-type pol β in mediating deletion of one or two TNRs during BER (Fig 4). We demonstrated that this was because the polymorphic variant exhibited similar capability to that of wild-type pol β of skipping over a small template loop structure during BER (Figs 5 and 6).

A previous study has shown that pol βR137Q exhibits 30% of wild-type enzymatic activity in the context of random sequence, which in turn reduces BER capacity and increases cellular sensitivity to alkylating DNA damaging agents as well as promotes apoptosis [64]. This effect has been further confirmed in a pol βR137Q transgenic mouse model *in vivo* in a recent study [79]. The results of the studies suggest that the pol β polymorphic variant exhibits significantly reduced DNA synthesis activity in random DNA sequence compared with wild-type pol β, and this may result in genome instability. However, our results showed that pol βR137Q polymorphic variant exhibited similar one-nucleotide gap-filling synthesis and strand displacement synthesis (S1 Fig) and slightly weaker DNA synthesis compared with the wild-type enzyme in the context of TNRs. Thus, pol βR137Q variant exhibited similar ability in skipping over a template loop (Fig 6) with the wild-type enzyme, thereby resulting in the same size of small repeat deletions as wild-type pol β. Since the dynamic TNR tracts readily form secondary structures such as hairpins and loops on the damaged and template strand, which would not form on random DNA sequence during BER [40, 44, 45, 71], Our results further indicate that secondary structures formed on the damaged and template strands of a TNR tract facilitated pol βR137Q to skip over a secondary structure on the template strand to readily perform DNA strand-displacement synthesis, thereby leading to the same effect on TNR instability as the wild-type enzyme.

A previous study has shown that pol βR137Q variant exhibits the impaired interaction with PCNA [64]. Our results showed that PCNA did not affect gap-filling synthesis activity of both wild-type pol β and pol βR137Q variant in the context of a TNR tract (Fig 2). This indicates that the impaired interaction between pol βR137Q variant and PCNA did not affect pol β activity and its resulted TNR instability during BER. However, our results cannot rule out a possibility that PCNA with post-translational modifications may alter the activity of wild-type pol β and pol βR137Q variant to modulate TNR instability during BER. Moreover, it is possible that the interaction between PCNA and other DNA polymerases can still modulate TNR instability by stimulating the activities of the DNA polymerases via cooperation with pol β DNA synthesis during BER. Previous studies have shown that ubiquitinated PCNA functions as a platform for polymerase switching between replicative DNA polymerases such as pol δ and translesion DNA polymerases such as pol η when encountered a DNA base lesion [80, 81]. Thus, it is conceivable that polymerase switching between replication DNA polymerases and translesion DNA polymerases that is mediated by ubiquitinated PCNA may occur when a hairpin or loop structure formed in the template strand during DNA replication and repair in the context of TNRs. It is also possible that PCNA-mediated polymerase switching between pol β and translesion DNA polymerases may occur during BER in a TNR tract in the postmitotic cells. It is of interest to further elucidate the roles of the interaction between ubiquitinated PCNA and pol β and its polymorphic variants as well as translesion polymerases in modulating TNR instability during BER.

In summary, in this study, we provided the first evidence that pol βR137Q polymorphic variant exhibited weaker DNA synthesis than wild-type pol β in the context of a TNR tract. We showed that the pol β polymorphic variant led to a weak FEN1 cleavage of TNR flaps compared with wild-type pol β during BER, and PCNA did not affect pol β DNA synthesis. Pol βR137Q polymorphic variant exhibited the same ability to skip over a template loop structure, thereby leading to the same TNR deletion as wild-type pol βduring BER. Since pol βR137Q variant exhibited the similar activity to that of wild-type pol β in the context of TNRs, our results suggest that human carriers of the pol β polymorphic variant may not exhibit a higher risk than the individuals bearing wild-type pol β in developing TNR expansion diseases.

## Supporting information

S1 Table Oligonucleotide sequences. (PDF)

S1 Fig Pol β R137Q variants exhibits similar gap-filling synthesis activity compared to that of wild-type pol β. Pol β gap-filling synthesis was conducted by incubating 0.1 nM, 0.2 nM and 0.5 nM of wild-type pol β or R137Q variant with 25nM ^32^P-labeled substrates (5’-end labeled). The experimental conditions are described in the Materials and Methods. **(A)** and **(B)** The gap-filling synthesis of pol β WT or pol βR137Q variant on the (CAG)_20_ substrates that contains a THF at the 5’-end or in the middle of the repeat tract. **(C)** and **(D)** The gap-filling synthesis of pol β WT or pol βR137Q variant on the (CTG)_20_ substrates that contains a THF at the 5’-end or in the middle of the repeat tract. Lanes 1 and 9 represent substrate only. Lanes 2 and 10 represent APE1 cleavage products. Lanes 3–5 and 11–13 represent pol β WT synthesized products. Lanes 6–8 and 14–16 represent pol β R137Q variant synthesized products. **(E)** The gap-filling synthesis of pol β WT or pol βR137Q variant on 1-nt gap substrate containing (CAG)_20_. (**F**) The gap-filling synthesis of pol β WT or pol βR137Q variant on the 1-nt gap substrate containing a random sequence. Lanes 1 and 8 represent substrate only. Lanes 2–4 and 9–11 represent pol β WT synthesized products. Lanes 5–7 and 12–14 represent pol β R137Q variant synthesized products. The red circles superimposed in the gels indicate the synthesized products of pol β WT or pol βR137Q.
(TIFF)

S2 Fig S1 nuclease digestion of the (CAG)_20_ substrates with a nick located after first CAG or after the tenth CAG. Both substrates were labeled at the 5’-end.of the template strand. (**A**) S1 nuclease digestion of the (CAG)_20_ substrate with a nick located after first CAG. Substrates were incubated with 2 U S1 nuclease. (**B**) S1 nuclease digestion of (CAG)_20_ substrate with a nick located after tenth repeat. Substrates were incubated with 5 U S1 nuclease. Lane 1 represents the substrate only. Lane 2 represents the reaction with APE1 alone. Lanes 3–7 represent reaction mixtures with S1 nuclease and APE1 at different time intervals. Lane 8 represents synthesized markers.
(TIFF)
