# Supplementary material for: Modulation of trinucleotide repeat instability by DNA polymerase β polymorphic variant R137Q
Source: PLoS One. 2017 May 5;12(5):e0177299. doi: 10.1371/journal.pone.0177299 (PMC5419657; doi:10.1371/journal.pone.0177299)
Supplement: S1 Table — (PDF) [file pone.0177299.s001.pdf]

S1 Table. Oligonucleotide Sequences

Oligonucleotide	nt	Sequence (5'-3')
**Undamaged strand**		
**CAG-0 (undamaged)**	100	CGA GTC ATC TAG CAT CCG TA CAG CAG CAG CAG CAG CAG CAG CAG CAG CAG CAG CAG CAG CAG CAG CAG CAG CAG CAG CAG TA CGT AGA CTT ACT CAT TGC
**CTG-0 (undamaged)**	100	GCA ATG AGT AAG TCT ACG TA CTG CTG CTG CTG CTG CTG CTG CTG CTG CTG CTG CTG CTG CTG CTG CTG CTG CTG CTG CTG TA CGG ATG CTA GAT GAC TCG
**THF containing substrate**		
**CAG-1 (THF 5'-end)**	99	CGA GTC ATC TAG CAT CCG TA CA**THF** CAG CAG CAG CAG CAG CAG CAG CAG CAG CAG CAG CAG CAG CAG CAG CAG CAG CAG CAG TA CGT AGA CTT ACT CAT TGC
**CAG-10 (THF middle)**	99	CGA GTC ATC TAG CAT CCG TA CAG CAG CAG CAG CAG CAG CAG CAG CAG CA**THF** CAG CAG CAG CAG CAG CAG CAG CAG CAG CAG TA CGT AGA CTT ACT CAT TGC
**CTG-1 (THF 5'-end)**	99	CGA GTC ATC TAG CAT CCG TA CT**THF** CTG CTG CTG CTG CTG CTG CTG CTG CTG CTG CTG CTG CTG CTG CTG CTG CTG CTG CTG TA CGT AGA CTT ACT CAT TGC
**CTG-10 (THF middle)**	99	GCA ATG AGT AAG TCT ACG TA CTG CTG CTG CTG CTG CTG CTG CTG CTG CT**THF** CTG CTG CTG CTG CTG CTG CTG CTG CTG CTG TA CGG ATG CTA GAT GAC TCG
**Template strand**		
**CAG-T (CAG-1 and CAG-10 template)**	100	GCA ATG AGT AAG TCT ACG TA CTG CTG CTG CTG CTG CTG CTG CTG CTG CTG CTG CTG CTG CTG CTG CTG CTG CTG CTG CTG TA CGG ATG CTA GAT GAC TCG
**CTG-1-T (CTG-1 template)**	100	GCA ATG AGT AAG TCT ACG TAC AG CAG CAG CAG CAG CAG CAG CAG CAG CAG CAG CAG CAG CAG CAG CAG CAG CAG CAG CAG TA CGG ATG CTA GAT GAC TCG
**CTG-10-T (CTG-10 template)**	100	CGA GTC ATC TAG CAT CCG TA CAG CAG CAG CAG CAG CAG CAG CAG CAG CAG CAG CAG CAG CAG CAG CAG CAG CAG CAG CAG TA CGT AGA CTT ACT CAT TGC
**1-nt gap substrate**		
**CAG-gap-upstream**	50	CGA GTC ATC TAG CAT CCG TA CAG CAG CAG CAG CAG CAG CAG CAG CAG CAG
**CAG-gap-downstream**	49	AG CAG CAG CAG CAG CAG CAG CAG CAG CAG TA CGT AGA CTT ACT CAT TGC
**CAG-gap-template**	100	GCA ATG AGT AAG TCT ACG TA CTG CTG CTG CTG CTG CTG CTG CTG CTG CTG CTG CTG CTG CTG CTG CTG CTG CTG CTG CTG TA CGG ATG CTA GAT GAC TCG
**Random-gap-upstream**	50	CGA GTC ATC TAG CAT CCG TA TCG CAC TGT TAT CAT TTC GTG TAC TTC ATG
**Random-gap-downstream**	49	GT ATG TGT CAT ATA TTC ATT TGC GCT AAC TA CGT AGA CTT ACT CAT TGC
**Random-gap-template**	100	5'- GCA ATG AGT AAG TCT ACG TAG TTA GCG CAA ATG AAT ATA TGA CAC ATA CAC ATG AAG TAC ACG AAA TGA TAA CAG TGC GAT ACG GAT GCT AGA TGA CTC G

**Nick substrate**		
**Nick-CAG-1-up**	23	CGA GTC ATC TAG CAT CCG TA CAG
**Nick-CAG-1-down**	77	CAG CAG CAG CAG CAG CAG CAG CAG CAG CAG CAG CAG CAG CAG CAG CAG CAG CAG CAG TA CGT AGA CTT ACT CAT TGC
**Nick-CAG-1-Template**	100	GCA ATG AGT AAG TCT ACG TA CTG CTG CTG CTG CTG CTG CTG CTG CTG CTG CTG CTG CTG CTG CTG CTG CTG CTG CTG TA CGG ATG CTA GAT GAC TCG
**Nick-CAG-10-up**	50	CGA GTC ATC TAG CAT CCG TA CAG CAG CAG CAG CAG CAG CAG CAG CAG CAG
**Nick-CAG-10-down**	50	CAG CAG CAG CAG CAG CAG CAG CAG CAG CAG TA CGT AGA CTT ACT CAT TGC
**Nick-CAG-10-Template**	100	GCA ATG AGT AAG TCT ACG TA CTG CTG CTG CTG CTG CTG CTG CTG CTG CTG CTG CTG CTG CTG CTG CTG CTG CTG CTG TA CGG ATG CTA GAT GAC TCG
**Primers for DNA fragment analysis**		
**CAG reverse primer**	23	**6-FAM** -CAA TGA GTA AGT CTA CGT ACT GC
**CAG forward primer**	21	CGA GTC ATC TAG CAT CCG TAC
**CTG-1 reverse primer**	23	**6-FAM** -CAA TGA GTA AGT CTA CGT ACA GC
**CTG-1 forward primer**	20	CGA GTC ATC TAG CAT CCG TAC
**CTG-10 reverser primer**	19	**6-FAM**- CGA GTC ATC TAG CAT CCG TAC
**CTG-10 forward primer**	21	GCA ATG AGT AAG TCT ACG TA
**Primers for site directed mutagenesis**		
**Pol beta R137Q fwd**	44	AAT TGA ACC ATC ATC AGC AGA TTG GG CTG A AAT ATT TTG GGG AC
**Pol beta R137Q rev**	44	GTC CCC AAA ATA TTT CAG CCC AAT CTG CTG ATG ATG GTT CAA TT
