# Supplementary figures and images for: Modulation of trinucleotide repeat instability by DNA polymerase β polymorphic variant R137Q
Source: PLoS One. 2017 May 5;12(5):e0177299. doi: 10.1371/journal.pone.0177299 (PMC5419657; doi:10.1371/journal.pone.0177299)

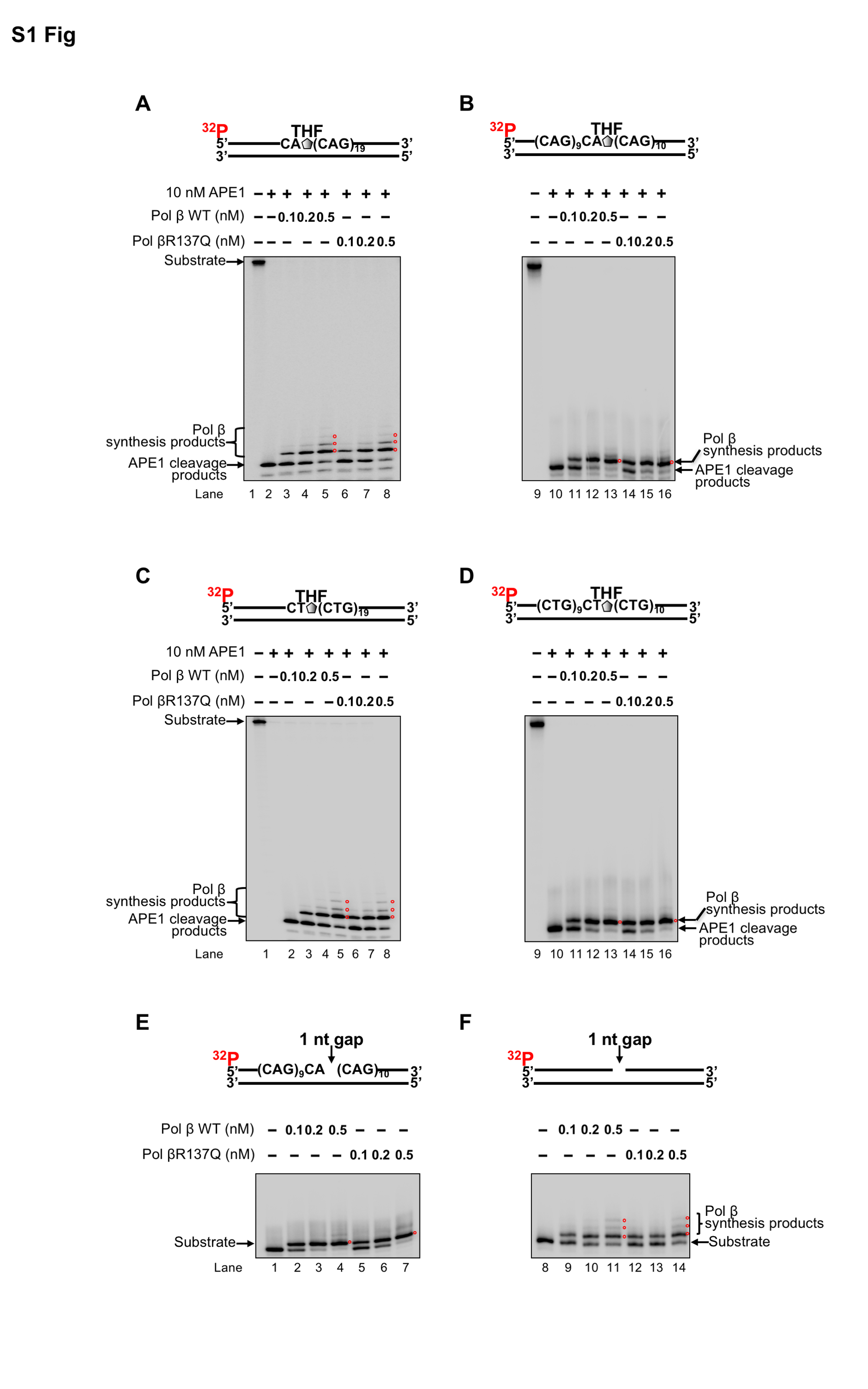

Supplement: S1 Fig — Pol β gap-filling synthesis was conducted by incubating 0.1 nM, 0.2 nM and 0.5 nM of wild-type pol β or R137Q variant with 25nM 32P-labeled substrates (5’-end labeled). The experimental conditions are described in the Materials and Methods. (A) and (B) The gap-filling synthesis of pol β WT or pol βR137Q variant on the (CAG)20 substrates that contains a THF at the 5’-end or in the middle of the repeat tract. (C) and (D) The gap-filling synthesis of pol β WT or pol βR137Q variant on the (CTG)20 substrates that contains a THF at the 5’-end or in the middle of the repeat tract. Lanes 1 and 9 represent substrate only. Lanes 2 and 10 represent APE1 cleavage products. Lanes 3–5 and 11–13 represent pol β WT synthesized products. Lanes 6–8 and 14–16 represent pol β R137Q variant synthesized products. (E) The gap-filling synthesis of pol β WT or pol βR137Q variant on 1-nt gap substrate containing (CAG)20. (F) The gap-filling synthesis of pol β WT or pol βR137Q variant on the 1-nt gap substrate containing a random sequence. Lanes 1 and 8 represent substrate only. Lanes 2–4 and 9–11 represent pol β WT synthesized products. Lanes 5–7 and 12–14 represent pol β R137Q variant synthesized products. The red circles superimposed in the gels indicate the synthesized products of pol β WT or pol βR137Q. (TIFF) [file pone.0177299.s002.tiff]

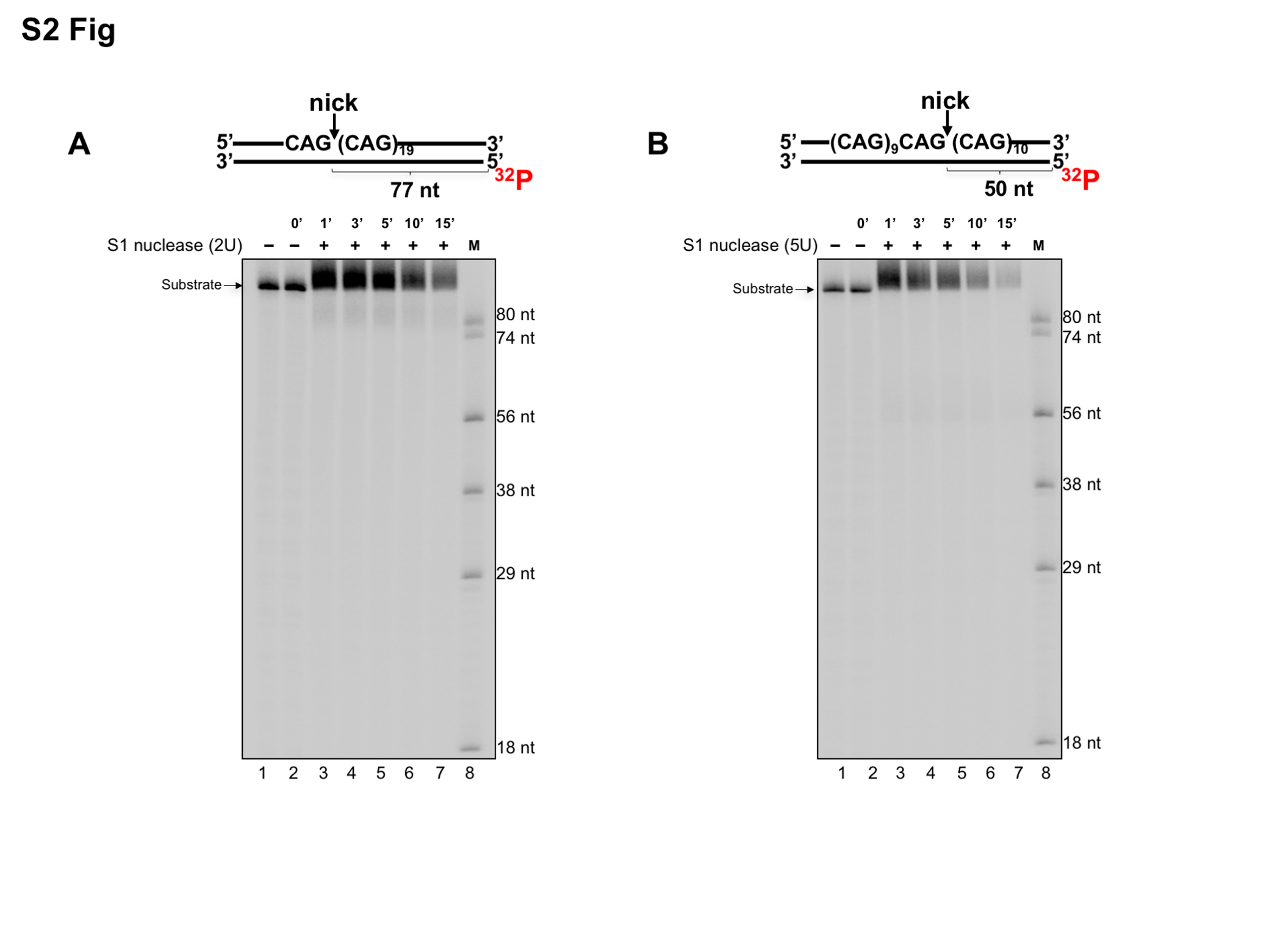

Supplement: S2 Fig — Both substrates were labeled at the 5’-end.of the template strand. (A) S1 nuclease digestion of the (CAG)20 substrate with a nick located after first CAG. Substrates were incubated with 2 U S1 nuclease. (B) S1 nuclease digestion of (CAG)20 substrate with a nick located after tenth repeat. Substrates were incubated with 5 U S1 nuclease. Lane 1 represents the substrate only. Lane 2 represents the reaction with APE1 alone. Lanes 3–7 represent reaction mixtures with S1 nuclease and APE1 at different time intervals. Lane 8 represents synthesized markers. (TIFF) [file pone.0177299.s003.tiff]
